# E-Cigarette-Associated Acute Severe Asthma in a Smoking-Naïve Adolescent: A Case Report

**DOI:** 10.7759/cureus.67422

**Published:** 2024-08-21

**Authors:** Lucas Küppers

**Affiliations:** 1 Institute of General Practice and Family Medicine, University Hospital Bonn, Bonn, DEU

**Keywords:** primary care, smoking prevention, health risks of e-cigarettes and vaping, severe asthma, e-cigarette use in adolescents, e-cigarettes

## Abstract

Electronic cigarettes (e-cigarettes) have become a prevalent phenomenon among adolescents and young adults, particularly as a perceived less harmful alternative to traditional tobacco cigarettes. A number of potential health risks associated with e-cigarettes have been identified, including links to cardiovascular diseases, asthma, and cancer. Given that adolescents have not yet completed their physical development, they are particularly susceptible to adverse health effects associated with e-cigarettes. This case report details the presentation of a healthy 16-year-old female patient who developed her first episode of acute severe asthma and a concomitant lower respiratory tract infection in a primary care practice setting. Prior to the onset of her symptoms, the smoking-naïve patient intermittently shared a nicotine-containing e-cigarette with a friend over a three-day period. Following outpatient treatment with inhaled corticosteroids, beta-2 agonists, and antibiotics, the asthma and lower respiratory tract infection were found to be reversible within the first week of treatment initiation. It is imperative that preventive measures at the political level be implemented to counteract the appeal and use of e-cigarettes among adolescents.

## Introduction

Since the advent of electronic cigarettes (e-cigarettes) in the 21st century, they have been promoted as a healthier alternative to tobacco cigarettes [1]. While countries such as the United Kingdom have distributed free e-cigarettes as a smoking cessation intervention [2], some countries such as India have completely banned the use and distribution of e-cigarettes [3]. Cross-sectional data from a study among adults in 14 countries revealed that 18.3 million individuals were current e-cigarette users [4]. A recent systematic review and meta-analysis by Salari et al. [4] found that current global e-cigarette use among adolescents is 4.8%, with males accounting for the majority of users [5]. In recent years, e-cigarettes have become a popular lifestyle product among adolescents and young adults. Hidden behind various flavors and liquids, harmful ingredients such as nicotine, vitamin E acetate, volatile organic compounds, and heavy metals pose a potential health risk to e-cigarette users [6]. Literature shows that e-cigarettes are associated with a variety of adverse health effects, including asthma, chronic obstructive pulmonary disease (COPD), lung cancer, and an increased risk of cardiovascular events [7]. Herein, we report a case of acute severe asthma in a smoking-naïve adolescent following intensive e-cigarette use over a three-day period.

## Case presentation

Case background

A 16-year-old female patient, accompanied by her parents, presented at a primary care practice on July 10 with a two-day history of progressive shortness of breath and a dry cough. She did not report any additional symptoms. The patient had no preexisting medical conditions and no history of allergic reactions. She had not undergone any surgical procedures and did not regularly take any medications. A review of the family history revealed no evidence of pulmonary disease. She denied the use of tobacco products. The patient was originally from another city and paid a visit to relatives.

Investigations

**Figure 1:**
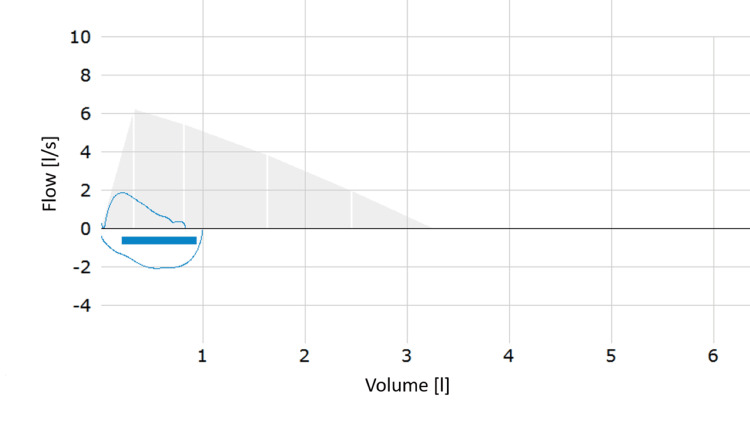
Initial PFT showing a flow-volume loop typical for obstruction with an FEV1 of 30%. PFT: pulmonary function testing, FEV1: forced expiratory volume in one second.

**Figure 2:**
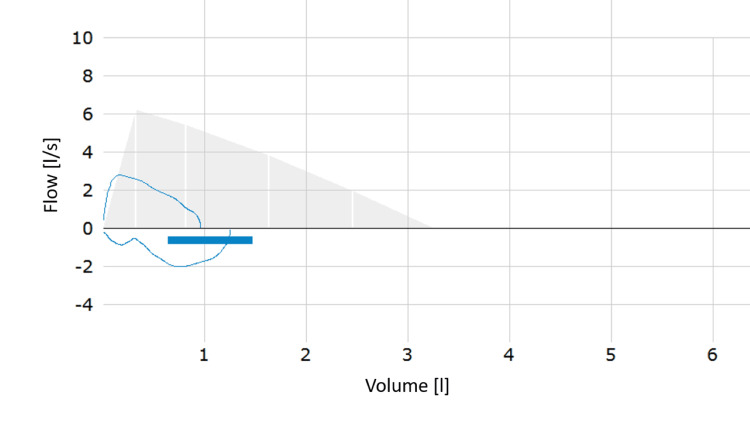
Second PFT after inhalation of SABA resulted in an increase of FEV1 to 35%. PFT: pulmonary function testing, SABA: short-acting β2-agonist, FEV1: forced expiratory volume in one second.

The physical examination revealed severe wheezing in both lungs, with a respiratory rate of 28 per minute. The patient exhibited tachycardia and a normal body temperature. Subsequent diagnostic procedures were conducted to ascertain the underlying cause of her respiratory distress. These included pulmonary function testing (PFT) and blood sampling to investigate the possibility of an infectious etiology. Due to her dyspnea, the patient was struggling to perform the PFT. The patient's peripheral oxygen saturation was 92%, and her forced expiratory volume in one second (FEV1) was reduced to 30% (Figure 1). Following the administration of two inhalations of 200 µg of a short-acting β2-agonist (SABA), a 16.7% increase in the FEV1 was observed, resulting in a value of 35% (Figure 2). Additionally, the patient reported a rapid onset of improvement in breathing. The saturation of peripheral oxygen increased to 96%. Based on the clinical findings and the results of the PFT, the patient met the criteria for the diagnosis of severe asthma in children aged five to 16 years according to the European Respiratory Society (ERS) guidelines [8]. A further anamnestic investigation, conducted in the absence of the parents, revealed that the patient had been celebrating her graduation from school, which occurred from July 5 to July 7. During this event, she used an e-cigarette for the first time by sharing it with a friend, having been persuaded to do so by her classmates. The refillable, battery-powered e-cigarette contained a nicotine-based liquid and was refilled once over a period of three days, with a total of two fillings consumed alternately by the patient and her friend. The initial onset of symptoms was observed on July 8, and the patient sought medical attention two days later.

Treatment and follow-up

**Figure 3:**
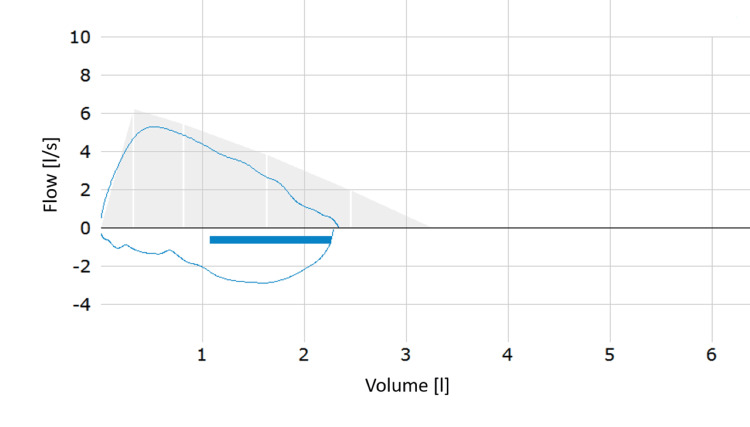
PFT after five days of treatment initiation showing an almost normal flow-volume loop with an FEV1 of 83%. PFT: pulmonary function testing, FEV1: forced expiratory volume in one second.

A hospital admission was proposed but declined by the patient and her parents, so the initial treatment regimen comprised a short-acting beta-2 agonist (SABA) as a demand inhaler medication and a fixed combination of an inhaled corticosteroid (ICS) and a long-acting beta-2 agonist (LABA). The patient was scheduled for regular outpatient follow-up visits, and in the event of an acute deterioration of clinical symptoms, an immediate presentation at the emergency room was recommended. On the following day, the patient's dyspnea had slightly improved, but a new onset of subfebrile temperatures was reported, which was confirmed on-site. The cough remained unaltered. The patient’s laboratory findings, received on the same day, indicated the presence of an acute infection with elevated leukocyte, thrombocyte, CRP, and neutrophil levels (Table 1). The patient apparently developed a concomitant lower respiratory tract infection (LRTI) concurrent with the onset of her asthma. The treatment regimen was extended to include amoxicillin 1,000 mg three times per day for one week due to the severity of asthma symptoms and elevated CRP levels with neutrophilia, which suggested the presence of a bacterial infection. Additional findings revealed the presence of a microcytic, hypochromic anemia and eosinophilia, the latter indicating the existence of an additional allergic component in the patient's asthma. On July 12, the patients’ subjective well-being improved. The saturation of peripheral oxygen was elevated to 97%, with both the respiratory and heart rates within the normal range. The patient exhibited no signs of wheezing upon auscultation of all lung sections. When the patient presented again on July 15, she reported no more dyspnea and a notable improvement in her general condition. The cough had almost completely ceased, and PFT showed an FEV1 of 83% (Figure 3). A smoking cessation intervention was conducted, explaining the adverse health effects of smoking. The patient credibly assured not to use any kind of e-cigarettes or other tobacco products in the future. As the patient resided in a different federal state and was required to return home, she was referred to a lung specialist in her place of origin for further diagnostic procedures and allergy testing. It was recommended that the actual therapy regimen be continued until further notice.

**Table 1:** Laboratory results indicating the presence of a bacterial infection.

Laboratory Test	Results	Reference Range
Leukocyte count /μL	14.4 +	5-10.5
Erythrocyte count x 10^6^/μL	4.78	4.1-5.1
Hemoglobin g/dL	9.3 -	11.5-16
Mean corpuscular volume fL	64.6 -	80-97
Mean corpuscular hemoglobin pg	20 -	28-33
Mean corpuscular hemoglobin concentration g/dL	30 -	33-36
Platelet count /μL	435 +	176-391
Absolute neutrophil count x 10^9^/L	11.63 +	2-7.5
Absolute lymphocyte count x 10^9^/L	1	1-3.2
Absolute monocyte count x 10^9^/L	0.97	0.4-1.3
Absolute eosinophil count x 10^9^/L	0.68 +	0.01-0.3
Absolute basophil count x 10^9^/L	0.1	0.01-0.2
C-reactive protein (CRP) mg/L	54 +	< 5

## Discussion

In the presented case, the patient received outpatient treatment, although it could be argued that she should have been admitted to the hospital. It is likely that the use of e-cigarettes resulted in a local inflammatory reaction, which subsequently led to the acute onset of severe asthma and facilitated an additional bacterial LRTI [9]. The case is consistent with the findings of recent literature. A systematic review by Li et al. revealed a clear correlation between the use of e-cigarettes and the onset of asthma in adolescents [10]. This correlation was demonstrated for current and even past use of e-cigarettes, indicating a long-term detrimental impact even after cessation of consumption [10]. While cases of smokers who developed adult-onset asthma under e-cigarette use have already been reported [11], this case underlines the heightened vulnerability of smoking-naïve adolescents due to the marketing-induced widespread misbelief that e-cigarettes are a less harmful alternative to combustible cigarettes [12]. In a cross-sectional study conducted in Australia, 26% of the 855 high school students surveyed indicated that they used e-cigarettes [13]. Half of them used the particularly addictive nicotine-containing e-cigarettes, which increase the brain’s susceptibility to addiction to other drugs [14]. This is in line with the results of a meta-analysis by Khouja et al., indicating that e-cigarette use of adolescent non-smokers might be associated with the initiation of smoking in later life [15]. As the case highlights, peer pressure also seems to play an important role as a socio-psychological component in adolescent e-cigarette consumers [16]. The dangers of e-cigarettes have garnered significant public attention since 2019, when several US citizens died from e-cigarette or vaping use-associated lung injury (EVALI). Since then, most cases of EVALI have occurred in adolescents and young adults [17]. Worryingly, the long-term health impacts of numerous e-liquid ingredients, including their potential to cause certain cancers, remain unknown until today [14]. It is important for primary care physicians who treat children and adolescents to be aware that the use of e-cigarettes is often concealed from parents. Empathetic communication including education and short interventions could be key here for the prevention and cessation of e-cigarette use [18]. On a macro-level, comprehensive preventive measures by health policies specifically addressing adolescents, young adults and schools should clarify the trivialization of e-cigarettes [19].

## Conclusions

E-cigarettes pose a significant health threat to adolescents and serve as an easily accessible gateway to tobacco product use. E-cigarette-associated severe asthma can occur with concomitant lower respiratory tract infection and appears to respond well to standard treatment. Health and education policymakers should be aware of the potential health risks posed by e-cigarettes and consider legal restrictions or bans on their distribution to prevent adverse health outcomes among younger users.

## References

[1] Kaisar MA, Prasad S, Liles T, Cucullo L (2016). A decade of e-cigarettes: limited research & unresolved safety concerns. Toxicology.

[2] Wilkinson E (2023). UK Government "swap to stop" plan to cut smoking rates. Lancet Oncol.

[3] Chakma JK, Kumar H, Bhargava S, Khanna T (2020). The e-cigarettes ban in India: an important public health decision. Lancet Public Health.

[4] Salari N, Rahimi S, Darvishi N, Abdolmaleki A, Mohammadi M (2024). The global prevalence of E-cigarettes in youth: a comprehensive systematic review and meta-analysis. Public Health Pract (Oxf).

[5] Pan L, Morton J, Mbulo L, Dean A, Ahluwalia IB (2022). Electronic cigarette use among adults in 14 countries: a cross-sectional study. eClinicalMedicine.

[6] Cao Y, Wu D, Ma Y (2021). Toxicity of electronic cigarettes: a general review of the origins, health hazards, and toxicity mechanisms. Sci Total Environ.

[7] Khanagar SB, AlBalawi F, Alshehri A (2024). Unveiling the impact of electronic cigarettes (EC) on health: an evidence-based review of EC as an alternative to combustible cigarettes. Cureus.

[8] Gaillard EA, Kuehni CE, Turner S (2021). European Respiratory Society clinical practice guidelines for the diagnosis of asthma in children aged 5-16 years. Eur Respir J.

[9] Kalininskiy A, Kittel J, Nacca NE, Misra RS, Croft DP, McGraw MD (2021). E-cigarette exposures, respiratory tract infections, and impaired innate immunity: a narrative review. Pediatr Med.

[10] Li X, Zhang Y, Zhang R, Chen F, Shao L, Zhang L (2022). Association between e-cigarettes and asthma in adolescents: a systematic review and meta-analysis. Am J Prev Med.

[11] Roberts J, Chow J, Trivedi K (2021). Adult-onset asthma associated with e-cigarette use. Cureus.

[12] Glantz SA, Nguyen N, Oliveira da Silva AL (2024). Population-based disease odds for e-cigarettes and dual use versus cigarettes. NEJM Evid.

[13] Leung J, Tisdale C, Choi J (2023). E‑cigarette use among high school students: a cross‑sectional study of associated risk factors for the use of flavour‑only and nicotine vapes. Int J Ment Health Addiction.

[14] Toll BA, Smith TT, King BA (2024). Nicotine e-cigarettes: considerations for healthcare providers. Nat Med.

[15] Khouja JN, Suddell SF, Peters SE, Taylor AE, Munafò MR (2020). Is e-cigarette use in non-smoking young adults associated with later smoking? A systematic review and meta-analysis. Tob Control.

[16] Murray JM, Sánchez-Franco SC, Sarmiento OL (2023). Selection homophily and peer influence for adolescents’ smoking and vaping norms and outcomes in high and middle-income settings. Humanit Soc Sci Commun.

[17] King BA, Jones CM, Baldwin GT, Briss PA (2020). The EVALI and youth vaping epidemics: implications for public health. N Engl J Med.

[18] Owens DK, Davidson KW, Krist AH (2020). Primary care interventions for prevention and cessation of tobacco use in children and adolescents: US Preventive Services Task Force Recommendation Statement. JAMA.

[19] Mylocopos G, Wennberg E, Reiter A (2024). Interventions for preventing e-cigarette use among children and youth: a systematic review. Am J Prev Med.

